# Alcohol-Induced Blackout

**DOI:** 10.3390/ijerph6112783

**Published:** 2009-11-06

**Authors:** Hamin Lee, Sungwon Roh, Dai Jin Kim

**Affiliations:** 1 Seoul St. Mary’s Hospital Department of Psychiatry, Catholic University of Korea, Catholic Medical Center / 505 Banpo-dong, Seocho-gu, Seoul 137-701, Korea; E-Mail: hamin@catholic.ac.kr; 2 Department of Mental Health Research, Seoul National Hospital / 51 Neungdong-ro, Gwangjin-gu, Seoul 143-711, Korea; E-Mail: swroh@korea.kr

**Keywords:** blackout, alcohol, memory

## Abstract

For a long time, alcohol was thought to exert a general depressant effect on the central nervous system (CNS). However, currently the consensus is that specific regions of the brain are selectively vulnerable to the acute effects of alcohol. An alcohol-induced blackout is the classic example; the subject is temporarily unable to form new long-term memories while relatively maintaining other skills such as talking or even driving. A recent study showed that alcohol can cause retrograde memory impairment, that is, blackouts due to retrieval impairments as well as those due to deficits in encoding. Alcoholic blackouts may be complete (en bloc) or partial (fragmentary) depending on severity of memory impairment. In fragmentary blackouts, cueing often aids recall. Memory impairment during acute intoxication involves dysfunction of episodic memory, a type of memory encoded with spatial and social context. Recent studies have shown that there are multiple memory systems supported by discrete brain regions, and the acute effects of alcohol on learning and memory may result from alteration of the hippocampus and related structures on a cellular level. A rapid increase in blood alcohol concentration (BAC) is most consistently associated with the likelihood of a blackout. However, not all subjects experience blackouts, implying that genetic factors play a role in determining CNS vulnerability to the effects of alcohol. This factor may predispose an individual to alcoholism, as altered memory function during intoxication may affect an individual’s alcohol expectancy; one may perceive positive aspects of intoxication while unintentionally ignoring the negative aspects. Extensive research on memory and learning as well as findings related to the acute effects of alcohol on the brain may elucidate the mechanisms and impact associated with the alcohol-induced blackout.

## 1. Introduction

Alcohol is a threat to global health, accounting for 4% of the global health burden, a proportion that is comparable to tobacco and hypertension [1]. Dysfunctions of multiple organ systems brought on by chronic alcohol use, including the brain, have long been the focus of medical concern, and are well documented in the public health literature. Nevertheless, alcohol continues to be a part of human culture. Acute effects of alcohol intoxication are a common, often voluntary experience and not necessarily considered a problem in itself. The alcoholic blackout, however, is one phenomenon of acute alcohol intoxication that merits special attention. Initial research in the 1950s reported that such blackouts are a hallmark of progressive alcoholism [2,3]. However, further research has proved otherwise, and blackouts are no longer considered as a signal of irreversible alcoholism [4]. Perhaps because the majority of people experiencing an alcoholic blackout are not clinically alcohol dependent, alcoholic blackouts may have been relatively neglected in terms of treatment intervention. Rather, legal matters have been the focus of cases where violations of the law are committed under intoxicated, amnesic states.

The mechanisms of an alcohol-induced blackout may be crucial in understanding its clinical implications. For a long time the effect of alcohol was thought to be a generalized depression of neural activity causing global impairment of cognitive, psychological, and behavioral domains [5–7]. An alcoholic blackout was perceived as the extreme manifestation of this effect. However, the blackout, characterized by amnesia during episodes of intoxication where the subject is conscious and able to carry on conversations or even drive a vehicle [8,9], is a manifestation of the selective effects of alcohol on specific brain systems. Previously, ethanol, a short chain lipid soluble compound, was thought to affect cells by a nonspecific lipid membrane disordering effect [5,6,10]. However, it is increasingly evident that alcohol interacts with specific neurotransmitter receptors, and current consensus is that specific regions of the brain are selectively vulnerable to acute effects of alcohol [5,10–15].

We need to make the memory staging terms clear as follows: immediate memory is also termed acquisition memory; short-term is also called retention memory; recent is called also consolidation memory and finally remote is referred to as also retrieval memory. Progress has been made in elucidating the mechanism of various memory systems and how they are affected by alcohol. There is increasing evidence that repeated alcoholic blackouts actually serve as a mechanism that facilitates alcoholism [2,16]. In this paper we aim to describe and discuss the alcoholic blackout to improve the understanding of this phenomenon and recognize its many faceted implications in medical practice and public health.

## 2. Methods

The PubMed database was searched (1965 to 2009) for epidemiological, pathophysiological, and social studies related to the alcoholic blackout, using the terms blackout, alcohol and memory, episodic memory, hippocampus and alcohol, and alcohol and the nervous system. The bibliographies of selected articles were used to extend the search. Articles were screened for their relevance to the specific topic of alcoholic blackout and related memory deficits on the basis of the title and abstract.

## 3. Clinical and Physiological Characteristics

### 3.1. Definition

An alcoholic blackout is amnesia for the events of any part of a drinking episode without loss of consciousness. It is characterized by memory impairment during intoxication in the relative absence of other skill deficits. It is not to be confused with ‘passing out’ [9]. Early documentation from Alcoholics Anonymous describes a variety of blackout behavior, especially in the en-bloc type, which includes driving for long distances or carrying on apparently normal conversations at parties. Subjects often report waking in strange places without any memory of how they got there. Criminal acts including murder, have been reported [17]. Although some have criticized these extremes, stating that such behavior is “exaggerated and a form of selective memory or denial to avoid guilt and confrontation over antisocial behavior brought on by drinking” [9], it nevertheless portrays the selective impairment of memory during an alcohol-induced blackout.

### 3.2. Types of Alcoholic Blackouts

An alcoholic blackout may be complete (en-bloc) or partial (fragmentary, or grayout) [2,9]. An en bloc blackout is complete amnesia for significant events otherwise memorable under usual circumstances. The defining characteristic of a complete blackout is that memory loss is permanent and cannot be recalled under any circumstances. Fragmentary blackouts occur more frequently [18,19]. In fragmentary blackouts, recall is usually possible and can be aided by cueing. Although initially the subject may be unaware that memory is missing, reminders usually help the subject remember forgotten events [9]. It is, however, difficult for investigators to be totally accurate because people may often fail to remember having a blackout, or do not attend to all circumstances in which they might have had a blackout, particularly fragmentary blackouts. Therefore, metamemory deficit is an issue in this type of research.

## 4. Epidemiology

A high frequency and volume of alcohol use is the single factor most closely related to experiencing blackouts [7]. In contrast to the older misconception that blackouts are an unlikely consequence of heavy drinking in nonalcoholics, anyone who drinks too much and too fast may experience a blackout [4]. For instance, 35% of trainees in a large pediatric residency program had reported experiencing at least one blackout [20]. In addition, 33% of first year medical students interviewed in another study acknowledged experiencing at least one blackout [8]. A survey of 2,076 Finnish males concluded that 35% experienced at least one blackout in the year before the survey [21]. Cultural and socioeconomic backgrounds are associated factors. The college campus is one subculture where excessive drinking is tolerated, if not encouraged. In a survey of 772 undergraduates, approximately one-half of those that had ever consumed alcohol reported experiencing at least one blackout during their lives, and 40% experienced one the year before the survey [22].

However in a four year follow up of young blackout drinkers, only 32% of respondents that were experiencing blackouts in the initial survey continued to experience them four years later. Alcoholic blackouts in this group appeared to have resolved spontaneously when the subjects graduated college, got married, or successfully entered the adult work force. Spontaneous resolution of blackout drinking appears to result from an interaction between informal support and objective social conditions such as full-time employment and a positive financial situation. To a certain extent, life transitional changes such as assuming adult roles appear to be a strong influence on the process of disengagement from problem drinking. Those who continued to experience blackouts after four years were male, comparatively young, unmarried, and with a lower socioeconomic status. The most salient predictor of chronic blackout drinking was the number of alcoholic relatives [9].

### 4.1. Risk Factors

Although a high blood alcohol concentration is required to induce a blackout, many drinkers reminisce that they have drank much more and not had a blackout [7]. A rapid rate of increase in blood alcohol concentration (BAC) is most consistently associated with the occurrence of an alcoholic blackout [7,23,24]. Therefore, gulping drinks, drinking on an empty stomach, or drinking liquor (opposed to beer) are risk factors of an alcoholic blackout [7]. However, not all subjects who drink rapidly and excessively experience blackouts, suggesting that there are individual that are genetically more vulnerable to alcohol-induced memory impairment [2,7,25]. Alcohol-induced blackouts are not necessarily the result of an underlying cognitive dysfunction; in a study of fragmentary blackouts, there was no baseline memory differences among the subjects that did or did not experience an alcohol-induced blackout [2]. This means that a baseline memory function does not seem a risk factor of blackouts.

## 5. Pathophysiology

A blackout is the result of alcohol-induced disruption of memory formation. The formation of memory involves the following processes: *encoding*, the initial registration and interpretation of stimuli; *storage*, consolidation and maintenance of encoded stimuli; and *retrieval,* which is the search and recovery of stored stimuli [2]. Alcohol has its greatest effects on encoding [26]. Short-term memory, which functions over a period of seconds, is relatively spared even during an en-bloc blackout, and recall of long-term memory, which applies on the scale of days to years, established before intoxication is also maintained [7].

Amnesia for events during intoxication involves impairment of episodic memory [3,7]. Episodic memory, by definition, includes the time, place, and other interrelated circumstances in which the event occurred. This contextual information is a prerequisite for formation of episodic memories [27,28]. Alcohol’s effect on encoding may disrupt the processing of context for the formation of an episodic memory. Because the episode was encoded with faulty context, free recall of this memory may be particularly difficult [29,30] or, depending on the degree of encoding impairment, even impossible, as in the case of en-bloc blackouts. In a fragmentary blackout, a striking feature is that cueing aids recall. Reminding a subject of events during the blackout often brings on more forgotten memories [4]. Such reminders, or cues, may provide contextual information during which a memory was formed, giving access to memory that was deficiently encoded.

The cellular mechanism by which a context is generated has been partially elucidated by observation of ‘place cells’ in rodents. Place cells are cells in the rodent brain which fire when the animal is in a particular location in the environment [31]. These location-specific cells ultimately create a spatial map in the brain, serving as a framework for event memories created in that environment. These cells are found in the CA1 pyramidal cell layer of the hippocampus. Alcohol profoundly suppresses activity of these cells. The dose-dependent suppression of CA1 pyramidal cells is consistent with dose-dependent effects of alcohol on episodic memory formation [28,32]. Evidence suggests that cognitive abilities mediated by the hippocampus might be particularly sensitive to the effects of moderate doses of alcohol [32]. In humans, hippocampal damage results in profound impairments in episodic memory with relative preservation of other functions in a way that is remarkably similar to an episode of an alcoholic blackout [10,31,33].

The molecular mechanisms of the effects of alcohol on the hippocampus are not clear. However, one leading candidate for a cellular substrate of memory formation is long-term potentiation (LTP), which is the establishment of long lasting heightened responsiveness to signals from other cells [7,34,35]. Alcohol inhibits establishment of LTP by potently antagonizing N-methyl-D-aspartate (NMDA) receptor activity [32,36,37]. The NMDA receptor is necessary for LTP induction in area CA1 of the hippocampus. Ethanol’s effect on LTP in area CA1 of the hippocampus is thought to involve both inhibition of the NMDA receptor and potentiation of the γ-aminobutyric acid A (GABA_A_) receptor transmission, which leads indirectly to further NMDA receptor inhibition [7,35,36].

Theories proposing that alcohol-related amnesia is a result of state-dependent effects of alcohol suggest that forgotten memories of events during intoxication may be recalled by returning to that intoxicated state [18]. However, in a study on fragmentary blackouts, participants that experienced fragmentary blackouts exhibited poor recall even after returning to an intoxicated state [38]. Although alcohol may act as a subjective, physiologic cue [2], a much more influential effect is its impairment of encoding [7,38].

Nevertheless, memory formation and retrieval are also influenced by other cognitive factors such as attention and motivation [39]. Some studies suggest that alcohol may have detrimental effects on certain aspects of retrieval [2]. A recent animal research paper showed that alcohol can cause retrograde memory impairments. Rats were allowed to learn while sober, but if that learning was followed by a very high dose of alcohol, then the next day or two they showed severe memory impairment. This suggests that blackouts are not always due to deficits in encoding, attention or other memory-related processes but also can be due to consolidation or retrieval impairments. The implication is that, for example, a person might be sober during an episode such as a conversation with someone, but then if this is followed by binge drinking this conversation might not be remembered even though there was no alcohol on board at the time. This retrograde amnesia was found to be prevented by caffeine and related agents, implicating the adenosine A2A receptor and phosphodiesterase [40]. The inconsistent study results underscore the need for further investigation to elucidate the role of alcohol in the development of blackouts.

## 6. Treatment Implications

Conventionally, an alcohol-induced blackout has been thought to be an essential early warning sign of problematic drinking, occurring very rarely in non-alcoholics. Previously, blackouts were ranked among the top three indicators of alcoholism, its course remaining relatively stable over time [3]. Although now it is clear that blackouts are not limited to alcoholics, it is a strong indicator of rapid and excessive drinking. A great majority of alcoholics experience blackouts during the early phase of addiction [41]. Even in young social drinkers, those that experience blackouts are characterized by more days of drinking, frequent heavy drinking, and a greater number of drinks per day. The influence of heavy drinking on the blackout incidence is even more compelling considering the fact that heavy drinkers are known to minimize self-reported estimates of drinking [9].

Although the alcohol-induced blackout itself may not be an indicator of progressive alcohol dependence, the way in which an individual views the experience of a blackout may be influential in determining future drinking behavior. Social learning theory implies that drinking patterns are maintained by biased beliefs about alcohol and one’s own behavior [2,42,43]. Among college students that experienced alcoholic blackouts, the majority was frightened by the amnesia and as a result decreased their intake of alcohol. Failure to appropriately modify drinking behavior after a blackout, in other words, chronic blackouts, may be a serious sign of alcoholism [4]. One’s drinking experience should play a role in determining one’s alcohol expectancy, but limited recall of events associated with intoxication may confuse one’s bases for outcome expectancies [2]. Alcohol’s initial effects are euphoria, which is then followed by more sedative effects [2,44,45]. It is reported that positive effects of alcohol occur more reliably among heavy drinkers [46] and that these positive effects occur at lower BAC [44]. Accordingly, positive expectancies are generally endorsed more strongly by heavy drinkers [47]. The variant aldehyde dehydrogenase 2 gene allele (ALDH2*2) is well known to be associated with negative physiological responses in normal samples in past research. A recent study showed, however, that the psychological expectancies associated with drinking are more positive and less negative in persons with alcoholism that have the ALDH2*1/*2 genotype [48]. This result implies that the positive expectancies associated with drinking alcohol appear to override the usual discomfort or negative effects associated with protection against drinking alcohol. Blackouts are associated with rising BAC, and recall of a drinking episode may reflect the initial positive effects better than the later negative effects. Those experiencing fragmentary blackouts have been reported to perceive a greater likelihood of positive alcohol effects compared to those who have not experienced blackouts, indicating that memory impairment during intoxication may produce a cognitive bias with regard to the alcohol associated experiences. In addition, those reporting en bloc blackouts had strong positive alcohol expectancies [2].

As mentioned above, the alcoholic blackout is a sign of brain dysfunction that results in memory impairment. In order to prevent alcohol-induced blackouts, the following is recommended: drink alcohol slowly, drink modestly, drink infrequently, drink with side dishes, and abstinence or moderation in drinking is especially important in high-risk groups, that is, persons with a large number of alcoholic relatives. Since alcoholic blackouts occur early in the course of disease and the blackout itself may act to facilitate problematic drinking resulting in another blackout, psychoeducation targeting episodes of alcoholic blackouts may be effective in preventing further episodes and the evolution to full-blown alcoholism.
